# Review Over a 3-Year Period of European Union Proficiency Tests for Detection of Staphylococcal Enterotoxins in Food Matrices

**DOI:** 10.3390/toxins8040107

**Published:** 2016-04-13

**Authors:** Yacine Nia, Isabelle Mutel, Adrien Assere, Bertrand Lombard, Frederic Auvray, Jacques-Antoine Hennekinne

**Affiliations:** Laboratory for food safety, ANSES, Université Paris-Est, F-94700 Maisons-Alfort, France; yacine.nia@anses.fr (Y.N.), isabelle.mutel@anses.fr (I.M.), adrien.assere@anses.fr (A.A.), bertrand.lombard@anses.fr (B.L.), frederic.auvray@anses.fr (F.A.)

**Keywords:** staphylococcal enterotoxins, Inter-Laboratory Proficiency Testing Trials, European Screening Method

## Abstract

Staphylococcal food poisoning outbreaks are a major cause of foodborne illnesses in Europe and their notifications have been mandatory since 2005. Even though the European regulation on microbiological criteria for food defines a criterion on staphylococcal enterotoxin (SE) only in cheese and dairy products, European Food Safety Authority (EFSA) data reported that various types of food matrices are involved in staphylococcal food poisoning outbreaks. The European Screening Method (ESM) of European Union Reference Laboratory for Coagulase Positive Staphylococci (EURL CPS) was validated in 2011 for SE detection in food matrices and is currently the official method used for screening purposes in Europe. In this context, EURLCPS is annually organizing Inter-Laboratory Proficiency Testing Trials (ILPT) to evaluate the competency of the European countries’ National Reference Laboratories (NRLs) to analyse SE content in food matrices. A total of 31 NRLs representing 93% of European countries participated in these ILPTs. Eight food matrices were used for ILPT over the period 2013–2015, including cheese, freeze-dried cheese, tuna, mackerel, roasted chicken, ready-to-eat food, milk, and pastry. Food samples were spiked with four SE types (*i.e.,* SEA, SEC, SED, and SEE) at various concentrations. Homogeneity and stability studies showed that ILPT samples were both homogeneous and stable. The analysis of results obtained by participants for a total of 155 blank and 620 contaminated samples allowed for evaluation of trueness (>98%) and specificity (100%) of ESM. Further to the validation study of ESM carried out in 2011, these three ILPTs allowed for the assessment of the proficiency of the NRL network and the performance of ESM on a large variety of food matrices and samples. The ILPT design presented here will be helpful for the organization of ILPT on SE detection by NRLs or other expert laboratories.

## 1. Introduction

Over the period 2010–2014, bacterial toxins represented the third most common causative agent of reported outbreaks within the European Union (EU), with staphylococcal enterotoxins (SEs) being considered as a major cause of foodborne illness due to bacterial toxins, *i.e.*, 49% of cases [1,2,3,4,5]. In fact, SEs produced by coagulase-positive staphylococci (CPS), including mainly *Staphylococcus aureus*, have super-antigenic and emetic activities, leading to toxic shock syndrome and staphylococcal food poisoning [6,7]. They are active in nanogram to microgram quantities, and are resistant to environmental conditions such as high or low temperature and pH that easily kill bacteria. Moreover, SEs are resistant to proteolytic enzymes, hence retaining their activity in the digestive tract after ingestion [8]. Thus, SEs are assumed to be a threat to public health, and notification of food poisoning outbreaks has been mandatory since 2005 [9].

Criteria for the enumeration of CPS and the detection of SEs in cheese have been set down in Commission Regulation (EC) No. 2073/2005 amended by Commission Regulation (EC) No. 1441/2007 [9] on microbiological criteria for food. This regulation defines process hygiene as well as food safety criteria. For milk and milk products, detection of SEs must be performed when the CPS count exceeds 10^5^ colony-forming units per gram (cfu/g). The confirmed presence of SEs in any foodstuff represents a potential hazard for human health as defined by Article 14 of Regulation (EC) No. 178/2002 [10]. On the other hand, the European Food Safety Authority (EFSA) indicated that 12 to 15 Member States (MSs) each year reported food poisoning outbreaks caused by SEs. From 2010 to 2014, the number of staphylococcal food poisoning outbreaks increased strongly, *i.e*., from 274 to 393. The most commonly reported food categories were mixed food (29.7%), meat and meat products (20.8%), cheese and dairy products (14.4%), bakery products (8.4%), and fish and fish products (6.5%) [1,2,3,4,5]. Even though the EC Regulation 2073/2005 defines a criterion on SEs only for cheese and dairy products, EFSA data highlights the involvement of other food categories, especially mixed food and meat products. 

Among the 23 SEs reported in literature [8,11,12], only five can be identified with commercially available immunoassay kits: SEA, SEB, SEC, SED, and SEE [13]. SEA is reported as the most frequently SE involved in staphylococcal food-poisoning outbreaks (SFPO) (80%) [14,15]. SED is another commonly detected SE; it can be quantified using an in-house quantitative ELISA method developed by the European Union Reference Laboratory (EURL) for CPS, in cheese and milk products, during both outbreak investigation and routine control analysis. SEC and SEE were also detected in outbreaks [16,17,18]. Immunological testing has become the method of choice for SEs identification due to the lack of another screening method which could be easily implemented and with available SE antibodies. Today, several ELISA kits are available for the detection of enterotoxins (SEA to SEE) in foods. Their specificity has not been studied extensively [19].

The Laboratory for Food Safety of the French Agency for Food, Environmental and Occupational Health & Safety (ANSES) has been appointed as EURL CPS, according to Commission Regulation (EC) No 776/2006 of 23 May 2006 [20]. In this context, it carries out reference activities for the Directorate General Health and Food Safety (DG SANTE) of the European Commission. The main tasks of EURL CPS in the field of SEs are (i) to select and/or develop analytical methods targeting SEs for the official control of milk and milk products and other matrices involved in SFPOs; (ii) to transfer those methods to National Reference Laboratories (NRLs); and (iii) to evaluate their ability to use the official method in order to comply with EU regulation towards official controls. The organization of Inter-Laboratory Proficiency Testing Trials (ILPTs) is thus a part of EURL activities, their objectives being (i) to verify the laboratories’ proficiency in implementing the official screening method for the detection of SEs in food and (ii) to ensure the reliability of the results obtained by the participating laboratories.

At the EU level, the official method for detecting staphylococcal enterotoxin types SEA to SEE in all categories of food is the European Screening Method (ESM) developed by the EURL CPS [13]. ESM is based on an extraction step with dialysis concentration followed by qualitative detection using either of two validated commercial assays, *i.e.*, immunoenzymatic Vidas^®^ SET2 (bioMérieux, Marcy l’étoile, France) and/or RIDASCREEN^®^ SET Total (R biopharma, Darmstadt, Germany) [21,22].

NRL participation in ILPTs is mandatory as part of their national reference mandate. Since validation of the ESM in 2011, EURL CPS organized three ILPTs (in 2013, 2014, and 2015) in which 31 NRLs representing 93% of the EU MSs participated.

The aim of this study was to evaluate the overall competency of the NRLs to implement the official method and to detect SEs in different food matrices. Eight food matrices representing the categories most commonly involved in SFPOs: cheese, lyophilized cheese, tuna, mackerel, ready-to-eat-food (a pie “Quiche Lorraine”, based on a mixture of bacon, butter, eggs, fresh cream, and milk), dessert cream, roasted chicken, and milk. They were spiked with four types of SE (SEA, SEC, SED, and SEE). The concentrations for each tested SE were selected based on those measured during investigation of SFPOs, routine and official control analysis performed in our laboratory. In addition, a naturally contaminated cheese was also used in the frame of the ILPT organized in 2013.

This article will describe the experimental design of European ILPTs, including sample preparation, homogeneity, and stability studies. The results obtained by participants and the performance of the EURL network over three years of SE detection in food matrices will be discussed.

## 2. Results and Discussion

Since 2001, EURL for CPS has been accredited (accreditation scope no. 1–2246 available at www.cofrac.fr) for SE detection in food products, according to Standard NF EN ISO CEI 17025 [23].This accreditation covers the use of ESM. Proficiency tests were performed according to the specifications of EN ISO IEC 17043 and ISO Guide 43 [24].

### 2.1. Proficiency Test Items and NRL Network Participation

Epidemiological data were investigated in order to determine the main food matrices, toxin types, and contamination levels involved in SFPOs, as well as the toxin types and the levels of contamination determined [1,2,3,4,5,6,18]. Thus, the capacity of the NRL network to detect SEs in food was assessed in eight matrices covering five food categories: ready to eat food, meat, milk products, pastry, and fish. SEA, SEC, SED, and SEE, identified in several food poisoning outbreaks in Europe, were selected for sample contamination. In the three ILPTs, a blank and two spiking levels were used (Section 2.2), each level being applied to the five food categories. Over three years, this ILPT scheme provided a precious additional data set to those formerly obtained during the validation study of ESM using a dialysis concentration step and both Vidas^®^ SET2 and RIDASCREEN^®^ SET Total kits [21,22].

Out of 29 European member states and associated countries, 28, 27, and 27 participated in the ILPTs dedicated to SE detection using ESM in food matrices organized in 2013, 2014, and 2015, respectively. Globally, 31 NRLs participated in the ILPTs, corresponding to a NRL participation rate of at least 86%.

### 2.2. Homogeneity and Stability Studies

As discussed in Section 4.4, quantitative criteria have been used for the assessment of homogeneity data. Depending on the concentrations of toxins used for sample preparation and on the resulting raw data, three contamination levels were distinguished, as follows:

Blank level, representing unspiked samples;
Level 1, representing samples contaminated at very low SE concentration associated with low raw data (test value (TV) or absorbance unit (AU) < 0.9);
Level 2, representing samples contaminated at low SE concentration associated with higher raw data (TV or AU > 0.9).

#### 2.2.1. Homogeneity Study

Based on qualitative criteria, SEs were not detected in 100% of the blank samples, and SEs were detected in 100% of the contaminated samples, regardless of the assay used (Table 1 and Table 2). Therefore, these samples were considered to be homogeneous for a qualitative analysis.

For the Vidas SET2 kit, mean values ranged from 0.52 to 0.76 TV and from 0.98 to 1.25 TV for levels 1 and 2, respectively. For the Ridascreen SET Total kit, mean values ranged from 0.28 to 0.88 and from 1.23 to 2.74 for levels 1 and 2, respectively. On the other hand, relative standard deviation (RSD) calculated for each couple food type/contamination level were less than 15%. Thereby, samples were considered to be homogeneous.

#### 2.2.2. Stability Study

**Figure 1:**
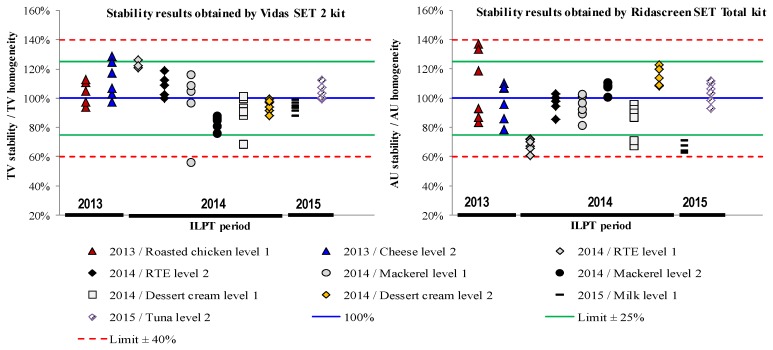
Results of the stability study. Comparison between data obtained after ILPT period (six replicates) and the assigned value obtained during the homogeneity study (*n* = 20).

Stability tests were performed after receiving all participants’ data in order to cover the entire ILPT analysis period; *i.e.*, 11, 9, and 8 weeks after sample dispatch for ILPT 2013, 2014, and 2015, respectively. Based on qualitative criteria, SEs were not detected in 100% of the blank samples (data not shown), and SEs were detected in 100% of the contaminated samples, regardless of the assay used (Figure 1). Thus, these samples were considered to be stable during each ILPT analysis period for a qualitative determination.

The six stability raw data obtained by each detection assay were compared to the assigned values (Equations 1 and 2). Figure 1 was obtained by dividing the value of each stability replicate by the mean of homogeneity values (in %).

(1) $$\;\frac{TV_{n\;stability}}{TV_{assigned}}\times 100\;\text{for Vidas SET2 assay}$$

where $TV_{n\;stability}$ is the test value of the *n*th replicate and $TV_{assigned}$ is the mean values obtained in the homogeneity study, which were thus considered as assigned values (Table 1).

(2) $$\frac{AU_{n\;stability}}{AU_{assigned}}\times 100\;\text{for Ridascreen SET Total assay}$$

where $TV_{n\;stability}$ is the test value of the *n*th replicate and $TV_{assigned}$ is the mean values obtained in the homogeneity study, which were thus considered as assigned values (Table 2).

For the Vidas SET2 assay, regardless of the food matrix, all replicate values were included in the interval of ±25%, except for one replicate of mackerel at level 1 (56%, ILPT 2014) and one replicate of dessert cream at level 1 (69%, ILPT 2014).

For the Ridascreen SET Total assay, most values were included in the interval of ±25%, except for ready-to-eat foods (RTE) at level 1, milk at levels 1 and 2, and replicate values of dessert cream at level 1, which were included in the interval of ±40%.

This quantitative assessment confirmed the qualitative results and the stability of samples over the ILPT analysis period.

### 2.3. EURL Network Results over a 3-Year Period

Most laboratories analyzed the ILPT samples according to ESM and returned their results before the deadline (except for one NRL during ILPT 2014). A few results were rejected when (i) the applicable version of ESM was not correctly performed; (ii) the NRL used none of the two validated detection kits; and (iii) the delay for analyses and/or for sending the results was not fulfilled.

After performing the extraction and dialysis concentration step, detection could be performed using either only Vidas SET2 or Ridascreen SET Total, or both kits. In this section, results obtained by each detection kit are assessed separately due to measures and instruments used for the two detection kits which are not comparable. Thus, the ability of the EURL network to perform each detection method can be evaluated.

For NRLs performing ESM with the Vidas SET2 detection kit, only 22 (3.6%) of the 615 samples sent to the NRLs over three years were rejected for data processing (Table 3). For NRLs performing ESM with the Ridascreen SET Total detection kit, only 6 (1.8%) of the 339 samples were rejected (Table 4).

Regarding blank and each contaminated level, only a few data have been rejected (≤4.4% for Vidas SET2 and ≤3.0% for Ridascreen SET Total). These observations indicated that the data obtained in the three ILPTs were considered to be significant for EURL network evaluation.

Table 5 and Table 6 show the results obtained by NRLs CPS on each type of food/contamination level over three years.

- For the blank level: 123 and 68 samples were analyzed using Vidas SET2 and Ridascreen SET Total kit, respectively. No negative deviation was obtained by NRLs, regardless of the detection kit used. Thus, ESM was considered as specific for SE in food matrices (100%).
- For level 1: 321 samples were analyzed using the Vidas SET2 detection kit and only two negative deviations were obtained on mackerel and cream dessert. For the Ridascreen SET Total kit, among the 169 samples, four negative deviations were obtained, on roasted chicken (two samples), cheese, and cream dessert. Thus, for level 1, the sensitivity of ESM was estimated at 99.3% and 97.6% for the Vidas SET2 and Ridascreen SET total, respectively.
- For level 2: among 171 samples, two negative deviations were obtained with the Vidas SET2 detection kit (RTE), and one negative deviation was obtained with the Ridascreen SET Total kit (mackerel). Thus, for level 2, the sensitivity of ESM was estimated at 98.8% and 99.0% for Vidas SET2 and Ridascreen SET total, respectively.
- Finally, trueness was estimated at 99.3% and 98.5% for ESM using Vidas SET2 and Ridascreen SET Total kits, respectively.

Overall, and taking into account the performance criteria on the qualitative results (specificity, sensitivity, and trueness), the EURL network for CPS obtained satisfactory results.

During the 2013 ILPT, 3.9% of results were rejected due to a deviation from ESM or non-respect of the organizers’ instructions. Also, 1.7% positive and/or negative deviations were obtained by participants. During the 2015 ILPT no deviation from the method nor from organizer’s instructions was reported (Table 7), indicating the efficiency of the measures implemented by NRLs after each ILPT. In fact, participants were able to describe any difficulties they encountered during testing and/or to add comments and observations. As a consequence, NRLs suggested some corrective actions in order to improve their reliability through technical exchange on ESM steps or by organizing training sessions, and these corrective actions were assessed by EURL. This task is part of reference activities requested by DG SANTE.

## 3. Conclusions

Even though the European regulation on microbiological criteria for food has settled a SE criterion only for cheese and dairy products, EFSA reported that various types of food matrices were involved in SFPOs. In this context, EURL network competency was evaluated for the first time through three ILPTs on a large panel of food matrices likely to be the source of SFPOs. A total of 31 NRLs participated to these ILPTs and analysed eight food matrices spiked with four types of SE (SEA, SEC, SED, and SEE) at different concentrations. Data assessment showed a significant progress of the EURL network proficiency. In fact, the rates of discrepancies identified decreased from 1.7% (ILPT 2013) to 1.0% (ILPT 2014), and finally to 0.0% (ILPT 2015).

The ILPT design presented in this work, having included a large panel of matrices tested, different types and concentrations of SE used, together with the homogeneity and stability studies, should be helpful for NRLs and other PT providers when organizing their own ILPTs.

## 4. Experimental Section

### 4.1. Sample Preparation

#### 4.1.1. Toxins

Highly purified freeze-dried SEs were purchased from Toxin Technology, Sarasota, FL, USA (batch no. 120794 A for SEA, no 113094C2 for SEC_2_, and no 70595E for SEE) and were rehydrated according to the manufacturer’s instructions to obtain stock solutions. Briefly, 1 mL of osmosis water was added to 1 mg of SE powder in order to obtain a theoretical concentration equal to 1 mg·mL^−1^. Purity has been checked for each toxin using SDS PAGE analysis.

#### 4.1.2. Preparation of the Proficiency Test Items

The three ILPTs were performed on eight matrices:

- Freeze-dried cheese samples (Tomme de Savoie) obtained from the Institute for Reference Materials and Measurements (EC/Joint Research Center/IRMM, Geel, Belgium).
- A raw cow’s milk cheese matrix (Bleu de Gex), naturally contaminated by SED (approximately 0.18 ng/g), from the sample collection of EURL CPS.
- Tuna, mackerel, ready-to-eat-food (pie, Quiche Lorraine), dessert cream (Crème brûlée, pastry), roasted chicken, and liquid semi-skimmed milk purchased from a retail store.

The SEs non-detection in a 25 g test portion was checked before sample contamination.

#### 4.1.3. Preparation of Blank and Contaminated Batches

Uncontaminated blank samples were homogenized and dispatched into flasks in order to obtain 25 ± 0.1 g.

Sample contamination was performed as follows. After homogenisation, 25 ± 0.1 g test portions were prepared in flasks and spiked separately by adding 500 µL of SE solution in PBS-BSA-Azide in each flask to obtain the target concentration (Table 8).

In order to prevent any cross contamination, each sample set of couple food/contamination level was prepared and contaminated separately. After their preparation, all samples were stored at −18 °C until homogeneity tests and shipment to participating laboratories.

#### 4.1.4. Identification of the Proficiency Test Items

The EURL guaranteed the full respect of confidentiality with regards to the identity of the participants in ILPTs.

In accordance with the internal ILPT Quality Manual, a random encrypted coding encompassing all samples was used. The samples were randomly coded independently of the laboratories’ codification to avoid any collusion with the results. The distribution of the samples within the different laboratories was also randomly performed.

### 4.2. European Screening Method for SE Detection in Food Matrices

This method includes an extraction-concentration step by dialysis and a detection step carried out using the Vidas SET2 (bioMérieux^®^, Marcy l’Etoile, France) or Ridascreen SET Total kit (R-Biopharm^®^ AG, Darmstadt, Germany), which are able to simultaneously detect SEA, SEB, SEC, SED, and SEE in food matrices [13].

Briefly, 25 ± 0.1 g of sample was mixed in 40 mL of distilled water at 38 ± 2 °C, using an Ultra Turrax homogenizer (T25-basic, Stanfen, Germany), and were shaken at room temperature for at least 30 min for toxin diffusion. In the case of liquid product, no distilled water was added.

Then, the pH of the slurry was adjusted to between 3.5 and 4.0 with HCl (Merck, Darmstadt, Germany) to precipitate caseins (in the case of dairy products) and centrifuged at 10,000× *g* at 4 °C for 15 min. The aqueous supernatant was sampled and adjusted to pH 7.5 ± 0.1 with NaOH (Merck) and centrifuged as above. The supernatant was filtered through glass wool and concentrated on a dialysis membrane with a molecular weight cut-off (MWCO) of 6000–8000 Da (Spectrum Laboratories Inc., Rancho Dominguez, CA, USA) against 30% (*w/w*) polyethylene glycol 20,000 (Merck, Darmstadt, Germany) overnight at 4 °C. The concentrated protein extract was recovered and adjusted to a final weight of 5.0 to 5.5 g using phosphate buffered saline (PBS: 145 mmol·L^−1^/10 mmol·L^−1^ NaCl/Na_2_HPO_4_, pH = 7.3 ± 0.2).

SE detection was performed from the extract using the two qualitative commercial assays (Vidas^®^ SET2 and/or the RIDASCREEN^®^ SET Total).

### 4.3. ILPT Design over 3 Years

Each year, 31 NRLs from 26 (27 for 2013) countries participated in the ILPT on SE detection in food matrices: AT, BE, BG, CY (2NRLs), CZ, DE, DK, ES, FI, FR, GR, HU, HR (1 NRL participated in ILPT 2013 and 2 NRLs in ILPT 2014 and 2015), IE, IT, LT, LV, MT, NL (2 NRLs), NO, PL (2 NRLs), PT, RO, SE, SI, SK (2 NRLs), UK (participated only in ILPT 2013). The list of participating NRLs is shown in Table 9.

Over three years, NRLs received eight food matrices spiked with various SE types at different concentrations levels. Homogeneity tests were performed before sample shipment and stability tests after receiving participants’ data.

Food matrices, spiking level, and number of replicates sent to the NRLs are reported in Table 8.

### 4.4. Data Processing

Vidas SET2 and Ridascreen SET Total are qualitative staphylococcal enterotoxin assays that should be used as primary screening tools. These detection assays are able to detect the presence or absence of five SEs (SEA to SEE) but they are not able to identify the SE type detected in the food extract.

Therefore, results obtained by participants were interpreted as “*SE not detected*” if raw data are below the positive threshold, and as “*SE detected*” if raw data are above the positive threshold. However, to assess results of the homogeneity study, the EURL added additional quantitative criteria (see Section 4.4.1) to better control the quality of the samples during the ILPT period.

#### 4.4.1. Homogeneity Study

According to the EN ISO 13528 Standard [25], the homogeneity study was performed on 20 flasks randomly sampled for each combination matrix/contamination level. Each sample was analyzed once on the same day according to ESM using both Vidas^®^ SET2 and RIDASCREEN^®^ SET Total kits. For blank samples, 100% of the obtained results must be below the positive threshold for the detection essay. For contaminated samples, 100% of the obtained results must be above the positive threshold for the detection kits and relative standard deviation (RSD) less than or equal to 15%. It should be noted that this RSD was calculated using the mean and the standard deviation of the 20 values.

#### 4.4.2 Stability Study

A stability test was performed after receiving all participants’ data. Six samples of each matrix/level were randomly selected and analyzed according to ESM. For the blank level, 100% of the obtained results must be below the positive threshold. For spiked levels, 100% of the obtained results must be above the positive threshold for the detection kits.

#### 4.4.3. Assessment of Participants’ Data

Only data obtained by participants using ESM were accepted and assessed. Results obtained by participants were interpreted as “*SE not detected*” or “*SE detected*” and compared with the expected results.

Accuracy of qualitative results was assessed according to the three following criteria:

**Specificity**: ability to obtain a negative response for a sample known not to contain any analyte

(3) $$Specificity=\;\frac{N^{-}}{N_{expected}^{-}}\times 100$$

where $N^{-}$ is the number of negative samples and $N_{expected}^{-}$ is the number of samples expected to be negative.

**Sensitivity**: ability to obtain a positive response for a sample known by the organizer to contain SE:

(4) $$Sensitivity=\;\frac{N^{+}}{N_{expected}^{+}}\times 100\;$$

where $N^{+}$ is the number of negative samples and $N_{expected}^{+}$ is the number of samples expected to be negative.

**Trueness**: ability to obtain a positive response for a sample known to contain SE and to obtain a negative response for a sample known to contain no analyte,

(5) $$Trueness=\;\frac{N}{N_{expected}}\times 100$$

where N is the number of samples correctly identified to be positive or negative and $N_{expected}$ is the total number of samples.

## Figures and Tables

**Table 1:** Homogeneity values obtained at each spiking level using the Vidas SET2 assay.

Spiking Level	ILPT Year	Food /Toxin	Vidas SET2 Kit	
			TV ^a^ (*n* = 20)	RSD %
**Blank**	2013	Roasted chicken	nd ^b^	/ ^c^
	2014	RTE ^d^	nd	/
	2014	Mackerel fish	nd	/
	2014	Cream dessert	nd	/
	2015	Freeze-dried Cheese	nd	/
**Level 1**	2014	Mackerel fish/SEC	0.52	9.2%
	2013	Roasted chicken/SEA	0.53	7.3%
	2015	Milk/SEA	0.55	4.4%
	2014	RTE/SEA	0.69	7.3%
	2014	Cream dessert/SEE	0.76	10.7%
**Level 2**	2015	Tuna fish/SEC	0.98	8.0%
	2014	Mackerel fish/SEC	1.01	8.0%
	2013	Cheese/SED	1.06	12.5%
	2014	Cream dessert/SEE	1.13	7.5%
	2014	RTE/SEA	1.25	6.7%

^a^ Test values; ^b^ Not detected; ^c^ Not relevant ^d^ Ready to eat food.

**Table 2:** Homogeneity values obtained at each spiking level using the Ridascreen SET Total assay.

Spiking Level	ILPT Year	Food/Toxin	Ridascreen SET Total Kit	
			AU ^a^ (*n* = 20)	RSD %
**Blank**	2013	Roasted chicken	nd ^b^	/ ^c^
	2014	RTE ^d^	nd	/
	2014	Mackerel fish	nd	/
	2014	Cream dessert	nd	/
	2015	Lyophilized Cheese	nd	/
**Level 1**	2013	Roasted chicken/SEA	0.28	13.0%
	2015	Milk/SEA	0.65	5.7%
	2013	Cheese/SED	0.66	13.3%
	2014	Cream dessert/SEE	0.73	14.8%
	2014	RTE/SEA	0.88	7.0%
**Level 2**	2014	Cream dessert/SEE	1.23	8.6%
	2014	Mackerel fish/SEC	1.42	12.2%
	2014	RTE/SEA	1.78	6.5%
	2015	Tuna fish/SEC	2.72	7.8%
	2014	Mackerel fish/SEC	2.74	9.9%

**^a^** Absorbance Unit; ^b^ Not detected; ^c^ Not relevant; ^d^ Ready to eat food.

**Table 3:** Rejected data and false results obtained by participants using Vidas SET2.

Spiking levels	Total Data	Rejected Data	Discrepancies ^a^
Blank	123	5 (4.1%)	0
Level 1	321	14 (4.4%)	2
Level 2	171	3 (1.8%)	2
All levels	615	22 (1.8%)	4

**^a^** negative and/or positive deviation.

**Table 4:** Rejected data and false results obtained by participants using Ridascreen SET Total.

Spiking levels	Total Data	Rejected Data	Discrepancies ^a^
Blank	68	1 (1.5%)	0
Level 1	169	5 (3.0%)	4
Level 2	102	0 (0.0%)	1
All levels	339	6 (1.8%)	5

**^a^** negative and/or positive deviation.

**Table 5:** Assessment of ILPT data obtained by national reference laboratories (NRLs) using Vidas SET2 over three years (2013 to 2015).

Years	Matrices	Type of SE	Spiking Concentrations (ng/g)	Spiking Levels	Vidas SET2 TV *	Number of Participants	Samples Analysed	Data Rejected	Positive Deviation	Negative Deviation	Accuracy		Trueness
											Specificity	Sensitivity	
2013	Roasted chicken	Blank	uncontaminated	Blank	0	23	23	1	0	-	100%	not applicable	99.30%
2014	Ready to eat food	Blank	uncontaminated		0	25	25	1	0	-			
2014	Mackerel fish	Blank	uncontaminated		0	25	25	1	0	-			
2014	Cream dessert	Blank	uncontaminated		0	25	25	2	0	-			
2015	Lyophilized Cheese	Blank	uncontaminated		0	25	25	0	0	-			
2014	Mackerel fish	SEC	0.150 ng/g	Level 1	0.52	25	75	3	-	1	not applicable	99.30%	
2013	Roasted chicken	SEA	0.028 ng/g		0.53	23	46	1	-	0			
2015	Milk	SEA	0.020 ng/g		0.55	25	50	0	-	0			
2014	Ready to eat food	SEA	0.055 ng/g		0.69	25	75	3	-	0			
2014	Cream dessert	SEE	0.150 ng/g		0.76	25	75	7	-	1			
2015	Tuna fish	SEC	0.250 ng/g	Level 2	0.98	25	50	0	-	0	not applicable	98.80%	
2014	Mackerel fish	SEC	0.250 ng/g		1.01	25	25	1	-	0			
2013	Cheese	SED	0.180 ng/g		1.06	23	46	0	-	0			
2014	Cream dessert	SEE	0.250 ng/g		1.13	25	25	1	-	0			
2014	Ready to eat food	SEA	0.110 ng/g		1.25	25	25	1	-	2			

* Test values.

**Table 6:** Assessment of ILPT data obtained by NRLs using Ridascreen SET Total over three years (2013 to 2015).

Years	Matrices	Type of SE	Spiking Concentrations	Spiking Levels	Ridascreen SET Total AU *	Number of Participants	Samples Analysed	Data Rejected	Positive Deviation	Negative Deviation	Accuracy		Trueness
			(ng/g)								Specificity	Sensitivity	
2013	Roasted chicken	Blank	uncontaminated	Blank	0	11	11	1	0	-	100%	not applicable	98.50%
2014	Ready to eat food	Blank	uncontaminated		0	15	15	0	0	-			
2014	Mackerel fish	Blank	uncontaminated		0	15	15	0	0	-			
2014	Cream dessert	Blank	uncontaminated		0	15	15	0	0	-			
2015	Lyophilized Cheese	Blank	uncontaminated		0	12	12	0	0	-			
2013	Roasted chicken	SEA	0.028 ng/g	Level 1	0.28	11	33	2	-	2	not applicable	97.60%	
2015	Milk	SEA	0.020 ng/g		0.65	12	24	0	-	0			
2013	Cheese	SED	0.180 ng/g		0.66	11	22	2	-	1			
2014	Cream dessert	SEE	0.150 ng/g		0.73	15	45	1	-	1			
2014	Ready to eat food	SEA	0.055 ng/g		0.88	15	45	0	-	0			
2014	Cream dessert	SEE	0.25 ng/g	Level 2	1.23	15	30	0	-	0	not applicable	99.00%	
2014	Mackerel fish	SEC	0.15 ng/g		1.42	15	15	0	-	1			
2014	Ready to eat food	SEA	0.11 ng/g		1.78	15	30	0	-	0			
2015	Tuna fish	SEC	0.25 ng/g		2.72	12	12	0	-	0			
2014	Mackerel fish	SEC	0.25 ng/g		2.74	15	15	0	-	0			

* Absorbance unit.

**Table 7:** Comparison between NRL results obtained each year.

Years	Samples Analysed	Data Rejected	Discrepancies
2013	181	7 (3.9%)	3 (1.7%)
2014	600	21 (3.5%)	6 (1.0%)
2015	173	0 (0.0%)	0 (0.0%)

**Table 8:** Design of the European Union Reference Laboratory (EURL) ILPT on the detection of SE in food matrices over three years (2013 to 2015).

ILPT Years	Food Matrix	Toxin	Spiking Concentration	Replicates per Participant	ILPT period		
					Analysis by Participants	Homogeneity Test	Stability Test
**2013**	Roasted chicken	uncontaminated	uncontaminated	1	From 19/11/2013 to 31/01/2014	08/11/2013	06/02/2013
	Roasted chicken	SEA	0.0175 ng/g	2		06/11/2013	06/02/2013
	Cheese	SED *	0.18 ng/g	2		30/10/2013	06/02/2013
**2014**	RTE **	uncontaminated	uncontaminated	1	From 13/05/2014 to 15/07/2014	09/04/2014	16/07/2014
	RTE	SEA	0.055 ng/g	3		12/03/2014	18/07/2014
	RTE	SEA	0.11 ng/g	1		14/03/2014	23/07/2014
	Mackerel fish	uncontaminated	uncontaminated	1		11/04/2014	16/07/2014
	Mackerel fish	SEC	0.15 ng/g	3		21/03/2014	18/07/2014
	Mackerel fish	SEC	0.25 ng/g	1		26/03/2014	23/07/2014
	Cream dessert	uncontaminated	uncontaminated	1		16/04/2014	16/07/2014
	Cream dessert	SEE	0.15 ng/g	3		28/03/2014	18/07/2014
	Cream dessert	SEE	0.25 ng/g	1		02/04/2014	23/07/2014
**2015**	Cheese	uncontaminated	uncontaminated	1	From 07/04/2015 to 31/05/2015	03/03/2015	02/06/2015
	Milk	SEA	0.020 ng/g	2		11/03/2015	02/06/2015
	Tuna fish	SEC	0.25 ng/g	2		06/03/2015	02/06/2015

* Naturally contaminated; ** Ready to eat food.

**Table 9:** List of NRLs participating in the ILPT dedicated to SE detection in food matrices from 2013 to 2015.

Country	Laboratory
Austria	AGES IMED/GRAZ
Belgium	Scientific Institute of Public Health—WIV-ISP
Bulgaria	National Center of Food Safety, NDRVMI Sofia
Croatia	Laboratory for Food Microbiology, Croatian Veterinary Institute
Croatia	Croatian national Institute of the Public Health
Cyprus	Laboratory for the Control of Foods of animal origin
Cyprus	State General Laboratory Cyprus/Food Microbiology Laboratory
Czech Republic	State Veterinary Institute Olomouc
Denmark	Danish Veterinary and Food Administration, division of microbiology
Finland	Finnish Food Safety Authority Evira
France	ANSES Laboratoire de sécurité des aliments—site de Maisons-Alfort/Unité SBCL, équipe Staphylocoques
Germany	NRL for CPS Germany—Federal Institute for Risk Assessment
Greece	Institute of Food Hygiene of Athens
Hungary	National Food Chain Safety Office, Food and Feed Safety Directorate, Food Microbiological National Reference Laboratory
Ireland	Dairy Science Laboratory
Italy	LNR per Stafilococchi coagulasi positivi incluso S. aureus—Istituto Zooprofilattico del Piemonte, Liguria e Valle d’Aosta
Latvia	Institute of Food Safety, Animal Health and Environment BIOR
Lithuania	National Food and Veterinary Risk Assessment Institute
Malta	Public Health Laboratory (Malta)
Norway	Norwegian Veterinary Institute
Poland	National Institute of Public Health—National Institute of Hygiene
Poland	National Veterinary Research Institute
Portugal	Instituto Nacional de Investigação Agrária e Veterinária, IP
Romania	Institute for Hygiene and Veterinary Public Health
Slovakia	National Reference Center of Environmental Microbiology
Slovakia	State Laboratory and Food Institute Dolny Kubin
Slovenia	Veterinary faculty, National veterinary institute, Institute for food hygiene
Spain	AECOSAN. Centro Nacional de Alimentación. Servicio de Microbiología Alimentaria
Sweden	National Food Agency
The Netherlands	Netherlands Food and Consumer Product Safety Authority
The Netherlands	RIVM, National Institute for Public Health and the Environment
United Kingdom	Gastrointestinal Bacteria Reference Laboratory
